# Development and Optimization Method for Determination of the Strawberries’ Aroma Profile

**DOI:** 10.3390/molecules29143441

**Published:** 2024-07-22

**Authors:** Iva Palac Bešlić, Martina Ivešić, Ivana Mandić Andačić, Danijela Bursać Kovačević, Irena Žuntar, Anica Bebek Markovinović, Fabijan Oštarić, Adela Krivohlavek

**Affiliations:** 1 Andrija Štampar Teaching Institute of Public Health, Mirogojska Cesta 16, 10000 Zagreb, Croatia; iva.palacbeslic@stampar.hr (I.P.B.); martina.ivesic@stampar.hr (M.I.); ivana.mandicandacic@stampar.hr (I.M.A.); 2 Faculty of Food Technology and Biotechnology, University of Zagreb, Pierottijeva 6, 10000 Zagreb, Croatia; danijela.bursac.kovacevic@pbf.unizg.hr (D.B.K.); anica.bebek.markovinovic@pbf.unizg.hr (A.B.M.); 3 Faculty of Pharmacy and Biochemistry, University of Zagreb, A. Kovačića 1, 10000 Zagreb, Croatia; irena.zuntar@pharma.unizg.hr; 4 Faculty of Agriculture, University of Zagreb, Svetošimunska Cesta, 25, 10000 Zagreb, Croatia; fostaric@agr.hr

**Keywords:** strawberry, solid phase microextraction, gas chromatography, aroma components

## Abstract

The strawberry (genus *Fragaria*) is a plant from the rose family (Rosaceae). As the fruits are likely to be picked mechanically, they are grown close to consumption centers. The aim of this work was to develop a suitable method for detecting as many molecules as possible in order to be able to distinguish between different strawberry cultivars and geographical origins in the future. Whole strawberries of the “Albion” cultivar, grown in the Jagodica Purgerica region of Zagreb, were used. Gas chromatography-mass spectrometry (GC-MS) in SCAN mode was used to analyze the aroma profile and to determine the proportion of individual components. The samples were prepared and analyzed using the solid-phase microextraction method (SPME). The impact of SPME fiber selection and GC column type was investigated, as well as sample weight, ionic strength, agitation temperature, and sampling time. A higher ionic strength was achieved by adding a 20% NaCl solution to the sample. The aroma profile of the studied strawberry cultivar consisted of furanone, esters, aldehydes, and carboxylic acids. Optimal results were achieved by adjusting the ionic strength during 15 min of extraction and incubation. The individual compounds were identified using NIST, Wiley libraries, and the “area normalization” method.

## 1. Introduction

Strawberries (*Fragaria* × *ananassa* Duch.) are one of the most consumed berries around the world. They are consumed fresh, but they are also very often used in the production of juices, jams, ice cream, and yogurt, as well as concentrated aroma preparations for domestic and industrial use [1]. It is worth mentioning that flavorings, natural and artificial, and certain food ingredients with flavoring properties for use in/on food in EU are regulated by Regulation (EC) No 1334/2008. Oral pharmaceutical products, OTC (over-the-counter) products, and dietary supplements also use approved and well-defined chemical substances that are thought to correlate positively with the intensity of strawberry flavor. These include linalool, ethyl butyrate, methyl butyrate, and 2,5-dimethyl-4-methoxy-3(2H)-furanone [2,3,4,5,6]. Strawberries are considered a low glycemic index fruit with bioactive compounds, vitamins, minerals, and antioxidant properties that may help in health maintenance and disease prevention, reducing the risk of serious health conditions like diabetes, cancer, stroke, and heart disease [7,8,9]. Strawberry is a widely studied fruit matrix whose aroma is the combined perception of many aromatic notes, such as fruity, sweet, caramel, and floral, and it has been studied worldwide [10,11,12,13]. The aroma components of different varieties of strawberries are very well presented in many studies [1,12,13]. More than 360 components that make up its characteristic smell and taste have been noted in previous studies [14]. Considering the complexity of the aromatic profile, the most common method of analysis is the headspace solid-phase microextraction method (HS-SPME). SPME was first developed approximately 25 years ago [15] and is currently the most widely used aroma extraction technique. SPME uses a coated silica fiber compound, mounted inside a syringe-like device [16].

Since consumer satisfaction depends on good nutritional quality resulting from taste and aroma, nowadays, the improvement of sensory properties is considered in breeding programs [17]. This paper aims to present a method for the analysis of the aromatic profile of strawberries and to optimize its parameters, which are considered to have the greatest influence on the final result of the analysis. The ultimate goal is an analysis of strawberries on the Croatian market using an optimized method, based on various analyses (stable isotope ratio and mineral composition analysis) connecting results with geographical origin, i.e., the authentication of Croatian strawberries. For this purpose, one type of strawberry (cv. “Albion”) from Croatia (Zagreb County) was used. It is called *Jagodica Purgerica*, famous for its quality and aroma, usually with no pesticide residue over the maximum residue level (MRL). It is harvested early in the morning, arrives at the stalls during the day, and is fresh and of good quality. It can be bought only in Zagreb County. Strawberries lose their nutritional value the longer they lie, so it is best to buy locally. Although aromatic profiling has been reported on many types of strawberries, including “Albion”, Croatian strawberries have not yet acquired their aromatic profile. The strawberries analyzed in this paper were grown without soil, using plastic bags filled with coconut fibers [18]. To determine the sensory quality of food, aroma is always on the list of valid parameters [14]. Aroma is different for different cultivars, but it also depends on storage conditions and maturity [19]. Aroma consists of many volatile compounds with different sensory characteristics. Chemical classes found in strawberries are mostly esters, as well as terpenes, furanone, and alcohols [20]. Esters are usually the most abundant [11], but other chemicals contribute to the odor and flavor of every strawberry [21]. The goal of this work is to create and optimize a method for aroma analysis. The aromatic profile contributes to the specificity of the fruit type and can be used in additional analyses to determine the geographical origin of the strawberry and distinguish between cultivars.

## 2. Results and Discussion

The flavor is characteristic of the species, consisting of many volatile carbohydrates (glucose, fructose), organic acids (citric acids), and non-characterizing volatile esters [22]. An individual fruit may have over a hundred different compounds that differ according to maturity [23]. Different studies have classified strawberry aroma as fruity, sweet, caramel-like, floral, and buttery [12,23]. According to Lewers et al. [24], who investigated consumer preferences for fresh strawberries, the “Albion” variety, along with Flavorfest and Allstar, was rated as significantly more acceptable in size (large enough) compared to other varieties in the study. Of the used cultivars evaluated in the aforementioned study, “Albion” received the highest rating for overall quality and ranked with the top cultivars of the 10. For overall quality, flavor, aroma, texture, and size, “Albion’s” high ranking was due to overall aroma, fruity aroma, and strawberry aroma with unremarkable sweetness and acidity [24]. In the past 30 years, various techniques have been used to study volatile compounds of several varieties of strawberry. More than 360 volatile compounds are reported as being related to its flavor [14]. Method parameters are critical steps for the reliable analysis of the molecules that are contained in the aromatic profile, and their influences have been investigated. Parameters such as extraction time and temperature, incubation time, and fiber coating were evaluated to optimize the extraction.

Although fiber can be immersed directly into a liquid sample for aroma extraction, it is more convenient to obtain an extract from the headspace above the food sample [25] because non-volatile materials in the food, especially lipids, can contaminate the fiber, therefore making it unusable. During SPME extraction, there is an equilibrium between the sample, the headspace, and the fibers for all volatile compounds in the sample.

### 2.1. Fiber and Column Polarity Effect

Influence of polarity on the aroma analysis was investigated by analyzing the strawberry samples on columns of different polarity with the associated SPME fiber. The polar analysis system includes an RXi Wax capillary column with a PDMS/CAR/DVB fiber, while the non-polar system includes a Rxi-5Sil MS column with a PDMS fiber. Both columns have dimensions of 30 m × 0.25 mm × 0.25 µm. The fiber was chosen according to the physical and chemical properties of the compounds expected (polarity, volatility) and considering a type of matrix [26]. As previously described by Drakula et al., PDMS is a non-polar coating resistant to high temperatures and effective in the extraction of non-polar compounds. CAR is a microporous coating with a large surface area which allows adsorption of trace volatile compounds and low molecular weight compounds [26], while DVB is a polar coating with characteristic macropores suitable for the extraction of partially volatile and polar compounds [26]. Food flavor is considered a complex matrix containing a large number of volatiles, and a large number of coelutions is a possibility both in polar and non-polar analysis systems, which may cause inaccurate identification of compounds [23]. Separation using the right column for compounds of different character, polarity, and chemical classes is important. Sanchez-Palomo et al. [27] tested three fibers in their study, analyzing volatile compounds in a skin sample of muscat grapes. In this study, compounds were expressed with area percentages. PDMS/DVB and CAR/DVB/PDMS fibers extracted a similar proportion of volatile compounds, mainly those responsible for the typical aroma of Muscat grapes (linalool, geraniol, and nerol), while with PDMS/DVB fiber, smaller RSDs were achieved.

Table 1 shows a list of compounds detected in the “Albion” strawberry by the SPME-GC-MS/MS method.

In our study, 50 analytes were identified on the polar column using PDMS/CAR/DVB, while 38 analytes were identified on the non-polar column using PDMS fiber. In the polar system, esters have a lower retention time, while terpene molecules have a higher retention time. In the non-polar system, terpenes are the most important compounds, i.e., terpenes have the biggest contribution to aroma profile (29%), followed by furans and derivatives (21%), while in the polar system, esters are the most abundant (42%). Many esters are detected in the polar system (Table 1) and they are most of the aroma profile. The most frequently found esters in the literature are the methyl and ethyl esters of hexanoic and butanoic acids [12,14,19,28,29,30,31].

According to Padilla-Jiménez et al. [32] and other authors [33] who investigated the “Albion” variety, esters are the dominant chemical class. Additionally, Padilla-Jiménez et al. concluded methyl butanoate increased in concentration during the maturation of “Albion” and Festival strawberries. The polarity of esters and their solubility in water depends on the length of the chain attached to the ester group. Esters with a lower molecular mass or with shorter chains have greater polarity and greater solubility in water. Esters with shorter chains are detected in the polar column (methyl butanoate and methyl hexanoate), while less polar esters (palmitate and mistyrate) are more likely to be detected in a non-polar column.

Another ester mentioned by several authors [33,34], methyl anthranilate, has an orange flower aroma and was not detected in our study. The reasons for this result may be that the ideal conditions of method analysis were not met, or this aroma compound is highly cultivar-specific [35]. The assumption that the “Albion” variety from Zagreb County does not contain this compound needs further investigation for a final conclusion. However, the most probable reason is methyl anthranilate being a “wild strawberry-like” [36] compound that is rarely contained in cultivated strawberries.

Lactones and terpenes can contribute to the pleasant coconut and citrus character in some cultivars [12] and contribute to the “sweet“ flavor. The lactones found on both columns in our study were gamma-dodecalactone and gamma-decanolactone. In the polar system, gamma-decanolactone has the largest peak area, thus having the greatest contribution to the aroma profile. This is not the case in non-polar systems, since PDMS is a less effective fiber, which agrees with previous studies [37]. Among terpenes, 10 of them were identified in the polar system and 14 in the non-polar system. Terpens detected only on the non-polar column were nerol, bisabol-12-ol, and bisabolol oxide. Monoterpenes (nerol, geraniol, linalool) and sesquiterpenes (bisabolene and famesene skeleton molecules) can be detected using both PDMS or PDMS/CAR/DVB fibers on the polar and non-polar column.

In non-polar systems, terpenes have smaller RT, nerolidol being the most abundant in both of them. L-alpha-terpineol, which is found in both systems, is a “wild strawberry-like” aroma monoterpene usually found in *Fragaria vesca* [34,36].

Four furan-related compounds have been detected in the polar system. Both compounds considered characteristic of strawberry [12,30], mesifuran (4-methoxy-2,5-dimethyl-3(2H)-furanone) and furaneol (2,5-dimethyl-4-hydroxy-3[2H]-furanone), were detected only on the polar column, while on the non-polar column, only 5-(1,5-dimethyl-1,4-hexadienyl)-2-ethenyltetrahydro-2-methyl furan was detected. Authors Kafkas E. et al. [1] detected furaneol when analyzing strawberries after liquid–liquid extraction with tert-butyl-methyl-ether, while they were not able to detect it with SPME extraction using PDMS/CAR fiber on 10 g of strawberry sample and added NaCl for ionic strength elevation. The authors used a non-polar column for SPME-GC/MS analysis, and this is probably the reason why furaneol was not detected by this technique. Its degradation depends on pH and temperature [37] and is also not stable in aqueous solutions [12,37]. On the other hand, even though these compounds have been identified in several studies, their concentration alters depending on strawberry variety, wild or cultivated strawberry, and maturity, and in certain cultivars, they are not detected at all [38].

In the polar column, three carboxylic acids were detected (decanoic, hexanoic, and butanoic acid), while not a single ketone was detected. In the non-polar column in the reverse case, four ketones were detected, but not a single carboxylic acid. Considering the polarity of these molecules, these results are expected.

It is generally accepted that the aroma compounds causing the smell or odor are part of the volatile fraction, whereas taste-active compounds usually belong to the nonvolatile food constituents [39].

Sheng et al. [34] connected aroma compounds with flavor: methyl butanoate and ethyl hexanoate have been described as having a pineapple aroma, gamma-decanolactone has been described as peachy, and methyl butanoate, hexanal, and hexyl acetate are considered fruity aroma type compounds. All these compounds except hexanal were found in the polar system analysis via applied method. Hexanal is one of the green odor compounds that are formed from linoleic acid by the alcohol dehydrogenase activity. Hexanal content is dependent on strawberries’ maturity and variety [40].

### 2.2. Sample Weight Influence

Most of the previous studies used pureed samples to analyze volatile compounds in strawberries, since every individual strawberry has to be cut and only part can be used for analysis [41]. Strawberry fruit consists of different tissues [42,43]. In this study, the strawberries were cut into quarters to imitate the state of eating a strawberry as closely as possible. Table 2 lists certain compounds with corresponding areas identified in strawberry samples under different conditions of SPME analysis.

**Figure 1:**
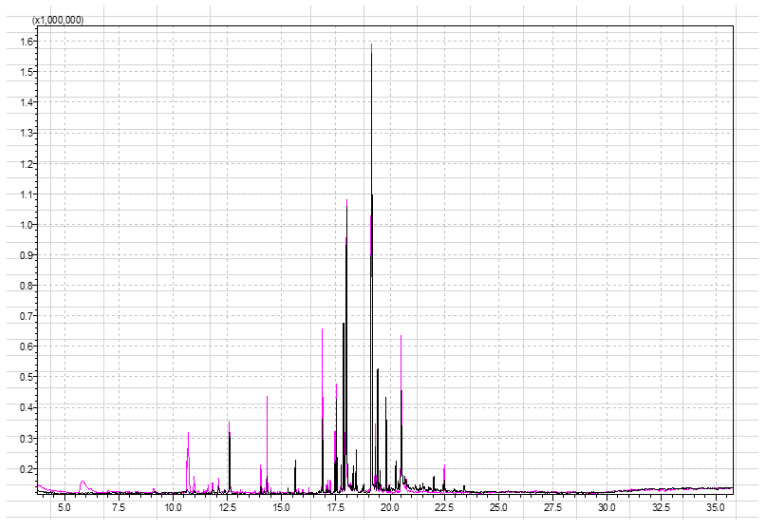
Superimposed chromatograms of strawberry samples obtained by performing the analysis under the same instrument conditions with 1 g and 5 g of sample for black and pink chromatograms, respectively.

Before investigating the most optimal method parameters for aroma analysis, the influence of the sample mass was investigated. For this purpose, the samples were weighed (1 g and 5 g were investigated) in glass headspace vials of 20 mL with a corresponding metal screw cap and a PTFE/silicone septum. Figure 1 shows a comparison of chromatograms obtained from analyzing strawberry samples of different weights (1 g of strawberries and 5 g of strawberries) under the same conditions (incubation at 60 °C for 15 min and extraction time of 15 min).

Figure 1 shows that 5 g of the sample gives a better response to certain signals. The first part of the chromatogram, which gives the response for the most polar compounds, is more visible in the pink chromatogram. Moreover, certain compounds have a response in the pink chromatogram while they do not have a signal in the black chromatogram, or their signal in the black chromatogram is so small that it is not possible to determine the identity of the analyte with sufficient probability. The significant difference occurs in the case of butyl hexanoate (at retention time 14.04 min), ethyl octanoate (at retention time 14.11 min), and octyl acetate (at retention time 14.33 min). Signals at this retention time belong to esters. Esters are the most abundant chemicals (25–90% of the volatiles) and provide the “fruity” and “sweet” odors [44]. According to the content of esters, different aroma groups may be classified. Another compound that appears in the pink chromatogram (mass of strawberry 5 g) is 2,5-dimethyl-4-methoxy-3(2H)-furanone (mesifurane). Mesifurane is a derivative of the compound 2,5-dimethyl-4-hydroxy-2H-furan-3-one and is very unstable under analytical conditions, which is why it is difficult to detect [44]. The analysis method used in this paper allowed the detection of mesifurane at retention time (RT) at 11.8 min. Table 2 (column 1 and column 2) lists compounds obtained from the SPME analysis of 1 g and 5 g strawberry samples.

In many studies, furanone is connected with flavor descriptions such as caramel-like, sweet, cotton candy-resembling, and fruity, and it might be considered the most important odor-active compound in strawberry aroma [13,24,38,45,46,47].

According to previous studies where the influence of sample weight was investigated, satisfactory results were obtained when analyzing a 10 g [1,12,42], 3 g [41], and 1 g sample [37]. Keeping in mind the higher intensity of short-chain esters, a mass of 5 g was taken as more optimal in this study. Both signal area and number of detected signals were higher in the 5 g sample analysis. Considering the significant difference in ester signal area, it can be concluded that the mass of the sample makes a difference. Although PDMS fiber was used, the 100 μm PDMS fiber is recommended by the manufacturer for the analysis of non-polar volatiles and has been wildly used for strawberry aroma analysis [28,48]. The influence of the selected fiber is explored below.

### 2.3. Ionic Strength Influence

**Figure 2:**
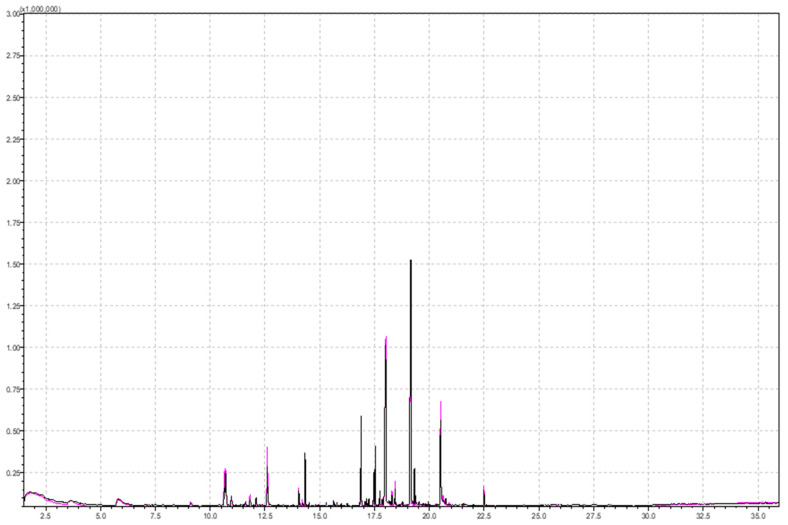
Superimposed chromatograms of a 5 g strawberry sample obtained by performing the analysis under the same instrumental conditions with added 5 mL of 20% NaCl solution and without NaCl solution for pink and black chromatogram, respectively.

Ionic strength has a great influence on the overall analysis, especially in the case of polar compounds, and increasing ionic strength is necessary for more efficient extraction of aroma components. A higher ionic strength in the sample was achieved by adding a sodium chloride (NaCl) solution to the strawberry sample, i.e., 5 mL of a 20% NaCl solution in water. Salt (NaCl or sodium sulfate, Na_2_SO_4_) addition increases the ionic strength of the solution, thereby reducing the solubility of the analytes and improving their volatility. Additionally, the extraction efficiency and the degree of adsorption to the fiber [49] were increased. Figure 2 shows a comparison of parts of chromatograms obtained by analyzing samples of different ionic strengths. The black chromatogram was obtained by analyzing the sample with the addition of NaCl solution, and a higher signal intensity can be observed. This is especially visible with compounds with shorter retention times (shorter RTs) and compounds of a higher polarity. These are short-chain esters (methyl butanoate, ethyl butanoate, methyl hexanoate, butyl butanoate, ethyl hexanoate, hexyl acetate) and also 2,5-Dimethyl-4-Methoxy-2,5-dimethyl-3(2H)-furanone (mesifuran), which is important to strawberry aroma (Table 2, column 2 and column 3).

Considering the difference in the total area of esters (1.5 times larger area with NaCl added), monoterpenes (1.7 times higher total area with NaCl added), and furans (1.8 times higher with NaCl added), ionic strength has a great influence on the analysis. Among monoterpenes, alpha-bisabolol and geraniol were detected in samples with higher ionic strength, which contributed to higher total area. Among furans, mesifuran was detected in samples with higher ionic strength, with an almost twice larger area (225,405 in sample with higher ionic strength versus 116,677 in sample with lower ionic strength).

The gamma-dodecalactone signal (RT = 20.5 min) has a slightly larger area in the chromatogram obtained from analyzing samples with the addition of NaCl.

Furthermore, 2-furanmethanol (furfuryl alcohol) was detected at RT 20.31 min when NaCl is added. This compound is water-soluble, and therefore, NaCl addition to the sample reduces its solubility, resulting in higher headspace concentrations. Furfuryl alcohol is next subsequently oxidized to furfural, which is present in fresh strawberries [50] but can also be the product of heat treatment in jam production [13]. Authors Oz et al. detected 2-furanmethanol (furfuryl alcohol) in Rubygem and Ventana strawberry varieties [51], while Ulrich and Olbricht [29] found 3-furanmethanol in sixteen analyzed cultivars. Al-Taher et al. detected 2-furanmethanol in freeze-dried strawberries, but not freeze-dried raspberries [37].

According to Vandendriessche and Nicolai [30], the addition of salt to aroma samples has been proven to inhibit enzymatic activity hydroxyperoxide lyase, which forms C6 aldehydes and increases when the fruit is wounded. This could be the reason for aldehydes appearing in chromatograms in strawberry aroma analysis (decanal). The addition of 1.0 M NaCl (0.5 mL:1 g) to blending does not alter the aroma composition [30]. Abouelenein et al. [13] concluded that it was better to work without the addition of NaCl salt because the addition of NaCl solution (they used 25% salt) increased the intensity of a few peaks but decreased others. Al-Taher and Nemzer [37] investigated the effect of adding an aqueous salt solution (20% Na_2_SO_4_) to the sample and concluded that a higher ionic strength was suppressed in the headspace of 10 analytes: oxime, methoxy-phenyl, nonanal, acetic acid, octyl ester, gamma-decalactone and gamma-dodecalactone, and butanoic ester and decyl ester. In our study, some compounds were also suppressed by ionic strength, mostly long-chain esters. Long-chain esters (octyl hexanoate, decyl-2-methyl butanoate, decyl isovalerate) are considered unpolar and are suppressed by adding NaCl to the sample. Increasing the ionic strength, the solubility of these compounds increased, resulting in smaller headspace concentrations.

### 2.4. Temperature and Time Influence

Extraction temperature and time are critical parameters in the SPME sampling process. Equilibrium during extraction depends on these parameters. Over time, analytes adsorb to the fiber, which increases the analyte concentration in the fiber until the equilibrium is reached. When equilibrium has been reached, the analyte concentration in the fiber decreases [27]. The extraction time affects the mass transfer of the volatile components to the fiber, while the temperature affects the kinetics of that process by creating vapor pressure of the components and encouraging diffusion [30]. Therefore, it is of great importance to extract at a proper temperature. High extraction temperatures can cause the decomposition or cooking of the sample. But higher temperatures may also be necessary when using the headspace technique, as it allows the extraction of higher boiling compounds. Increasing the temperature facilitates the transfer of the analyte from the solution to the headspace above the solution, thus accelerating the extraction of the analyte, but this procedure reduces the sensitivity of the method [52]. While the parameters of the GC and MS part of the method were constant, the agitator temperature, the incubation time, and the extraction time were varied to find the most optimal parameters. The extraction time was observed at 15 min and 30 min at three different temperatures: 40 °C, 60 °C, and 80 °C.

#### 2.4.1. Incubation Temperature

The analytical technique used for aroma analysis is important, since it affects the abundance of the compounds detected. SPME and/or HS coupled with GC is more efficient for the ester analysis, whereas liquid–liquid extraction procedures coupled with GC downsize the contribution of esters and increase the abundance of carboxylic acids and furanones [53]. An incubation temperature of 40 °C proved to be the least effective for the extraction of aroma molecules.

**Figure 3:**
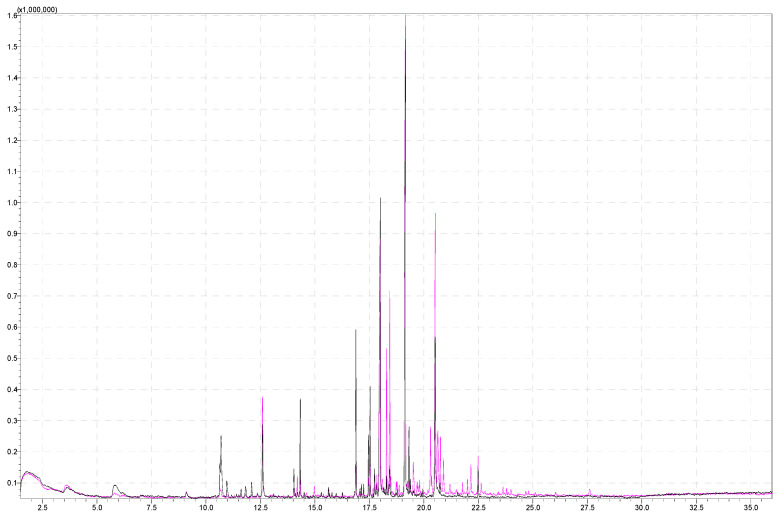
Superimposed chromatograms of a strawberry sample obtained by performing the analysis under the same instrument conditions, analyzing 5 g of strawberries at 80 °C and 60 °C agitator temperatures for the pink and black chromatograms, respectively.

Figure 3 shows a comparison of parts of the chromatograms obtained by analyzing a sample of 5 g of strawberries at different agitator temperatures, 60 °C and 80 °C. The intensity of individual signals is higher at a temperature of 80 °C (pink chromatogram). Only increasing the incubation temperature (from 60 °C to 80 °C) has a positive effect on the analysis, imparting signals that were not detected at a lower temperature. Additionally, Table 2 (column 2 and column 3) represents signal areas at the temperatures of 60 °C and 80 °C and the aroma compounds obtained from SPME analysis of strawberry samples. Individual signal areas are higher with increased incubation temperature, but the chromatogram at the lower incubation temperature is lacking certain compounds important for aroma analysis, such as short-chain esters (methyl-butanoate at RT 3.65 min, ethyl-butanoate at RT 5.81 min). The chromatogram at the higher incubation temperature is also lacking certain compounds important for aroma analysis (methyl hexanoate at RT 9.10 min, hexyl acetate at RT 10.96 min, butyl isovalerate at RT 11.61 min). Certain compounds that occur at both incubation temperatures but have larger areas at higher incubation temperatures are butyl-butanoate at RT 9.09 min and linalool at RT 12.61 min. Mesifuran has a higher intensity at a lower incubation temperature with a higher sample weight, as well as in the case of a smaller sample weight but higher ionic strength.

Furthermore, furan (5-(1,5-dimethyl-1,4-hexadienyl)-2-ethenyltetrahydro-2-methyl-furan) eluted at a retention time of 18.3 min and detected with similarity index 83% is present in both chromatograms, with a larger area at a higher temperature. The mentioned compound has a retention time of 18.3 min and 18.4 min because it appears as stereoisomers [54] (cis and trans) and was extracted from a smaller number of strawberry varieties (Ventana and Fortuna) [51]. This compound is floral-scented and is detected in *Brunfesia grandiflora* [55] and matured tequila [56].

Sesquiterpene alcohols, such as alpha-bisabolol at RT 20.65 min and farnesol at RT 20.91 min, were detected at higher incubation temperatures but not at lower incubation temperatures. In contrast, bisabolol was detected with a lower incubation temperature (60 °C) but higher ionic strength. Our aroma analysis showed farnesol to be a compound constituent, which is not in accordance with the previously published data of Oz et al. [51] They stated that farnesol was the most abundant alcohol in both Rubygem and Ventana strawberries but was not identified in “Albion” varieties.

Compounds that are of particular interest in strawberries include terpenes, since they have characteristic sensory properties and are known to have potential antimicrobial activity [57]. It is known that among these compounds, volatile monoterpenes (C10) and sesquiterpenes (C15) were identified in strawberries and other fruits [13]. Furaneol has a higher boiling point at 188 °C [58] and is expected to give a higher signal at higher temperatures when analyzing the aroma profile, but it was not detected in our study, most likely due to polarity issues. This is a polar compound, and PDMS fiber is not appropriate for its detection.

Monoterpenes, nerol, and geraniol were detected at higher incubation temperatures and were not detected at an incubation temperature of 60 °C.

At RT 20.53 min, compound gamma-dodecalactone was eluted, which gives a 2.17 times larger peak area at a higher incubation temperature of 80 °C (area 2,661,323 vs. area 1,222,723). The γ-dodecalactone plays a major role in the fruity aroma and the overall aroma of the strawberry [34].

**Figure 4:**
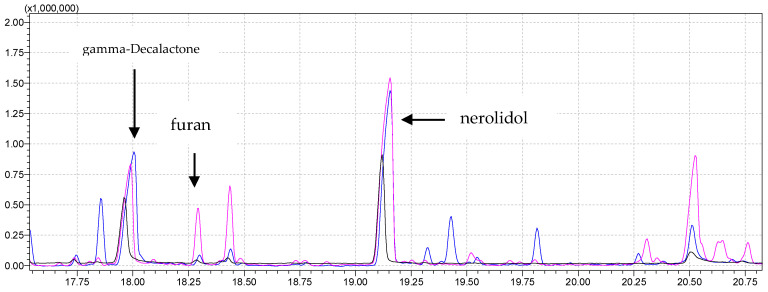
Superimposed parts of chromatograms of a strawberry sample obtained by performing the analysis under the same instrument conditions by analyzing 1 g of strawberries with no NaCl added at 80 °C, 60 °C, and 40 °C agitator temperature for the pink, blue, and black chromatograms, respectively.

Figure 4 shows a comparison of the chromatogram analysis of a sample of 1 g of strawberries at all three different incubation temperatures. Generally speaking, esters’ boiling points are lower than those of carboxylic acids and alcohols of similar molecular weight because there are no intermolecular hydrogen bonds between ester molecules.

The total area of detected esters at an incubation temperature of 80 °C was greater than at a lower temperature (0.40 times greater). Individual esters were more abundant at a lower temperature (octyl acetate, decyl acetate, octil-2-metylbutanoate, octyl-3-metylbutanoate). On the other hand, at a higher temperature, other esters were detected (methyl butanoate, ethyl butanoate, hexyl hexanoate).

Ruiz et al. [49] found that increasing the extraction temperature (from 40 °C to 60 °C) increased the amount of aldehydes, acids, and high-molecular weight alcohols while decreasing the amount of ketones and low-molecular weight alcohols extracted.

#### 2.4.2. Incubation Time

**Figure 5:**
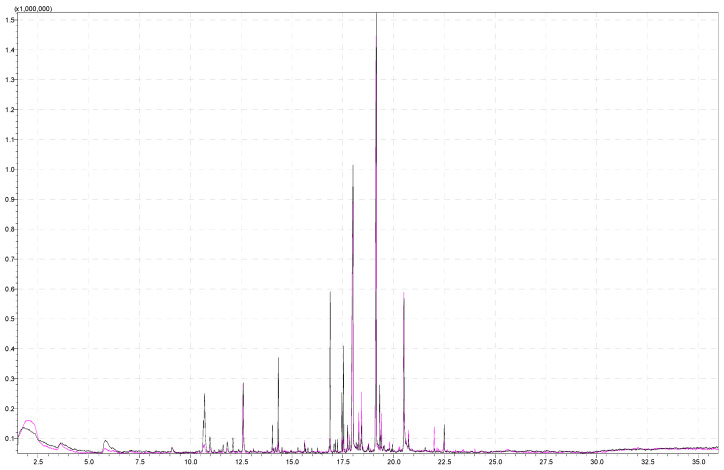
Superimposed chromatograms of a strawberry sample obtained by performing the analysis under the same instrument conditions by analyzing 5 g of strawberries at 15 and 30 min incubation times for the black and pink chromatograms, respectively.

To investigate the impact of incubation time on aroma analysis, 5 g of sample was weighted to a headspace vial, and 5 mL 20% of NaCl solution was added. The temperature of the agitator was kept at 60 °C. Figure 5 shows a comparison of chromatograms obtained at different incubation times (15 min and 30 min). Signals that have a higher intensity at an incubation time of 15 min represent esters, whose intensity decreases with a longer incubation time. The same was already observed in earlier studies [59]. Moreover, mesifuran, which is characteristic of strawberries, has a smaller peak area at a longer incubation time. On the other hand, Zhang et al. [60] investigated the influence of incubation time and temperature and concluded that both the total peak area and the number of identified volatile compounds gradually increased with incubation time. According to Zhang et al. [60], extraction efficiency increased with temperature and time and was the highest under the conditions of 80 °C incubation temperature/30 min time. Table 2 (column 4 and column 5) lists the majority of analytes detected at both incubation times.

The most noticeable difference in total areas was in the case of esters (2.8 times higher area for a lower incubation time). At a lower incubation time, individual esters were more abundant (for example, methyl butanoate, ethyl butanoate, ethyl hexanoate, octyl-2-methylbutanoate, octyl-3 methyl butanoate, and hexyl acetate). The signal area of mesifuran was higher at lower temperatures, and the other two furans were more abundant at lower incubation times. It is apparent that equilibrium was reached at a lower temperature.

#### 2.4.3. Extraction Time

**Figure 6:**
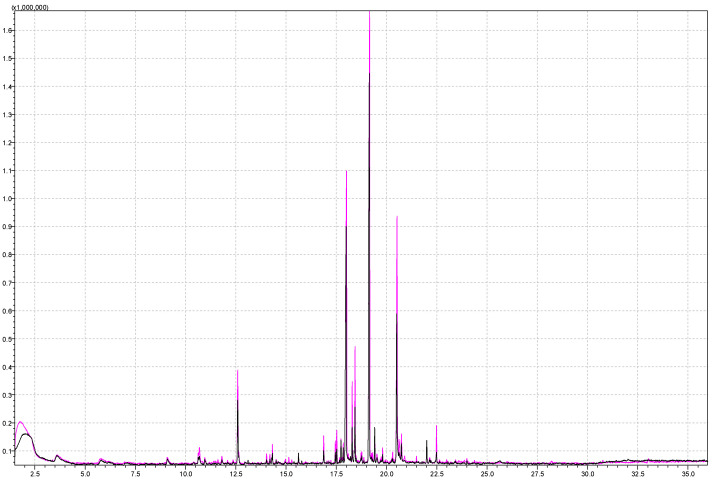
Superimposed chromatograms of a strawberry sample obtained by performing the analysis under the same instrument conditions by analyzing 5 g of strawberries at 15 min and 30 min extraction time for the black and pink chromatogram, respectively.

Extraction is the progression of analytes achieving equilibrium between the sample matrix, headspace, and the coating of the fiber. The incubation time was kept at 30 min and the incubation temperature at 60 °C, while the extraction time was variable. Figure 6 shows the chromatograms of the strawberry aroma analysis at different extraction times of 15 min and 30 min. According to the figure, it is evident that the intensities of the majority of signals are higher at 15 min extraction time. In both chromatograms, the most common signals for the strawberry aroma are identified (methyl butanoate, ethyl butanoate, methyl hexanoate, butyl butanoate, ethyl hexanoate, hexyl acetate, 3(2H)-4-methoxy-2,5-dimethyl furanone). A significant difference in the signal area was found with these compounds: octyl acetate (63,368 on 15 min extraction time vs. 124,460 on 30 min extraction time), octyl-3-methylbutanoate (87,736 at 15 min extraction time vs. 192,827 at 30 min extraction time), and gamma-dodecalactone (1,247,452 at 15 min extraction time vs. 2,395,865 at 30 min extraction time).

With increasing extraction time, decreases in peak area were found for methyl hexanoate (145,866 for 15 min extraction time and 181,137 for 15 min extraction time). A study by Howard et al. [61] investigated the connection between SPME extraction and the optimum time to hold the fiber in the headspace of the sample (extraction time). They concluded that if the extraction time was too long, competition for sites on the fiber could cause inaccuracies in the relative amounts of analytes present. On the other hand, if the extraction time was shortened, it may be insufficient to achieve an equilibrium between the three phases in the SPME analysis: the aqueous sample solution, the vapor phase, and the fiber. In this study, 30 min was determined to be the optimum extraction time. Furthermore, according to Q.L. Ma et al. [62], an increase in the peak area was found, mostly for the less volatile compounds, at higher temperatures and with a longer extraction time. Longer extraction times did not have much effect on the low-boiling point volatile compounds, such as 3-methylbutanal and methylpyrazine, according to the same authors. In this study, at 30 min extraction time, 5-(1,5-dimethyl-1,4-hexadienyl)-2-ethenyltetrahydro-2-methyl-furan was not detected, and nor were individual esters (nonyl acetate, decyl acetate). The total areas of monoterpenes and sesquiterpenes were higher with increased extraction time, but alpha-bisabolene was not detected with increased extraction time.

Table 2 (column 5 and column 6) shows a list of compounds obtained in SPME analysis of strawberries’ samples at different extraction times.

**Figure 7:**
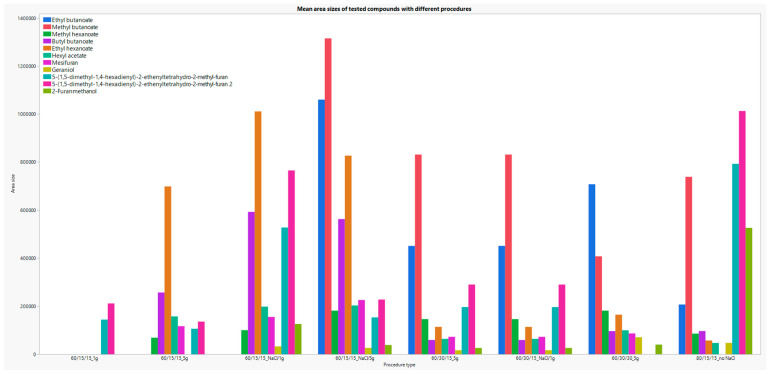
Graphical representation area dependency of the selected analytes on the parameters of the SPME method.

Figure 7 shows the dependence of certain analytes on method parameters that varied during optimization. Analytes that showed great sensitivity to incubation or extraction temperature and/or time of the sample (ethyl butanoate, methyl butanoate, methyl hexanoate, butyl butanoate, ethyl hexanoate, hexyl acetate, mesifuran) and analytes that were previously rarely detected in strawberry samples (5-(1,5-dimethyl-1,4-hexadienyl)-2-ethenyltetrahydro-2-methyl- furan, 2-Furanmethanol) are shown graphically.

### 2.5. Area Normalization

The main purpose of this study was not to determine compound quantity, since accurate quantification of analytes requires the use of a certified reference standard for each identified compound. Considering that dozens of VOCs may be present in the sample, their quantification based on the inclusion of standards for each VOC increases the economic costs considerably. Additionally, the problem is that adequate standards may not be available for most compounds [63].

In our study, the simplest method for normalizing GC-MS data, total sum normalization (TSN), was used. In brief, the amount of all identified compounds for each sample was set as 100%. The area of each peak in a profile was divided by the total sum of all peaks to determine the contribution of a particular compound in the sample in the form of a percentage [64]. This method of normalization was chosen since the aim of this study was not the exact quantification of individual analytes, but the investigation of the influence of individual parameters of the analysis on the overall composition of aromas. Also, the aim was to check which component of the strawberry’s aromatic profile contributes the most to the overall aromatic profile and which components are an important part of the whole aromatic profile.

The amount of individual components was shown in the form of a percentage obtained by the method of area normalization according to the relation [64] (1):

(1) $$x_{ij}^{TSN}=\frac{x_{ij}}{\sum_{i=1}^{n}x_{ij}}*100$$

$x_{ij}$ represents the area of the selected compound, while $\sum_{i=1}^{n}x_{ij}$ represents the sum of areas of all identified compounds in the sample. The ratio of these quantities was multiplied by one hundred, giving $x_{ij}^{TSN}$ as result, which represents the percentage of an individual component within the aromatic profile of the sample regarding the total composition of the identified compounds. The combination of identifications based on the use of commercial databases and quantifications according to chosen internal standards or comparison of their retention indices (RIs) with the published data was performed by many authors [11,65,66,67,68]. The exact concentration of compounds is demanding and exhaustive, since several dozen or even hundreds of compounds can be detected in different concentrations [39]. Terpenes occur as stereoisomers, which creates more problems for identification and quantification. Aroma compounds have never been analyzed by the standardized methods described in the Metabolomics Standards Initiative (MSI). Considering 670 entries for strawberries, it is obvious that the methodology for aroma analysis should include the development and exchange of reproducible and falsifiable data [69]. The exact quantitation in GC is only possible through coelution of isotope-labeled references (stable isotope dilution analysis) or using the standard addition method [36]. Achievement of these conditions was described in papers by Schrieble and Hofmann [11] and Li et al. [67].

## 3. Materials and Methods

### 3.1. Plant Material

Strawberry cultivar “Albion”, named as Jagodica Purgerica, was grown in the greenhouse of a private company in Donja Lomnica, Zagreb, Croatia, in 2021 [18]. The planting was carried out during November 2021; the strawberries were grown on coconut fibers. The fruits were harvested in June 2022 at 2 levels of ripeness: 75% (ripe) and 100% (fully ripe) [18]. After picking, each sample was stored in a freezer at a temperature of −20 °C. In this study, the whole fruits were used to set up and optimize the method for aromatic component profile analysis.

### 3.2. GC-MS Analysis

The prepared samples were analyzed with a conventional gas chromatograph coupled with Shimadzu GC-MS-TQ8050 NX mass spectrometry and an AOC 6000 automatic sampler (Kyoto, Japan). The instrument was equipped with a software package for starting the instrument and processing data, GCMS Solution and LAB Solution Insight (Shimadzu Corporation, Kyoto, Japan). The analytes were chromatographically separated on a Rxi-5Sil MS capillary column and a WAX capillary column. Both columns had dimensions of 30 m length × 0.25 mm inner diameter × 0.25 µm film thickness and were manufactured by Restek, Bellefonte, PA, USA. The samples were analyzed in SCAN mode according to the parameters shown in Table 3. The analytes were identified by comparing their mass spectra with those stored in NIST (National Institute of Standards and Technology, Gaithersburg, MD, USA) and Wiley (Hoboken, NJ, USA, SAD) libraries.

### 3.3. SPME Analysis

Two types of fibers were used: PDMS (Polydimethylsiloxane) and DVB/CAR/PDMS (Divinylbenzene/Carboxen/Polydimethylsiloxane). Each fiber was conditioned according to the manufacturer’s instructions (Supelco Inc., Bellefonte, PA, USA). A comparison of the chromatograms obtained by extraction on individual fibers was made. To optimize the parameters of the method, SPME extraction was performed at different incubator temperatures (60 °C and 80 °C), different incubation times (15 min and 30 min), and different extraction times (15 min and 30 min). The desorption time was kept at 10 min. Method parameters are listed in Table 4. Process efficiency was evaluated by considering the total number of compounds extracted and their peak areas. The samples were prepared in glass headspace vials of 20 mL with a corresponding metal screw cap and a PTFE/silicone septum (Precision Labware, Arlington, TX, USA).

### 3.4. Sample Preparation

Sample preparation is an important step in aroma analysis. HS-SPME is a simple and efficient, solvent-free sample preparation method suitable for the analysis of volatile aromas. Each sample was prepared by cutting the strawberry fruit into quarters, and these parts of the fruit were weighted directly into the headspace vials using tweezers. To establish the influence of ionic strength on aroma analysis, 5 mL of 20% sodium chloride (NaCl) saturated aqueous solution was added to chosen samples to reduce the solubility of the required compounds. Each sample was closed in a 20 mL vial with an aluminum cap and a silicone-PTFE septum. Each experiment was performed a minimum of 2 times.

### 3.5. Graphical Presentation

The parameter distributions were tested, and a graphical representation for area dependency of the selected analytes on the parameters of the SPME method was plotted with a probability level of 0.05 in JMP ^®^ Pro, Version 16, SAS Institute Inc., Cary, NC, USA, 1989–2023 [70].

## 4. Conclusions

In general, ionic strength and sample mass had the most effect on aroma analysis. According to the results obtained in our study, the optimal conditions for analyzing the aromatic profile of strawberry, cultivar “Albion”, Jagodica Purgerica, are 60 °C incubation temperature/15 min incubation time/15 min extraction time. These parameters are optimal to achieve equilibrium. Furthermore, the optimal sample weight is 5 g with mandatory ionic strength adjustment by adding a 20% NaCl solution. With the selection of optimal analysis conditions, it is possible to analyze strawberries using the SPME method in a polar as well as non-polar system. Fiber that is more suitable for aroma analysis is PDMS/CAR/DVB fiber. Polar column analysis identified the compounds previously described as the most important for strawberry aroma, such as esters, mesifuran, and furaneol. Our study concluded that the Croatian Jagodica Purgerica of the “Albion” variety is notable for its aromatic profile and thus its quality. The next step in the research of strawberries in the Zagreb area is the quantification of individual analytes according to an optimized method and the comparison of their composition with strawberries from other parts of Croatia or the world in order to achieve the desired goal: determining the geographical origins and molecules that would be considered fingerprints for different regions or variants. To confirm the results obtained in this study, further analysis is needed using both Rxi-5Sil MS and WAX capillary columns, using certified reference material or retention indices that should be calculated for each compound by comparing them to the retention times of alkanes in alkane mixture C8-C20.

## Figures and Tables

**Table 1:** Compounds detected in “Albion” strawberry analyzed by SPME-GC-MS/MS.

Compound	RTX-Wax Column and PDMS/CAR/DVB Fiber	RTX-5Sil Column and PDMS Fiber
	AREA %	AREA %
**Esters**		
Methyl butanoate	1.94	ND *
Ethyl butanoate	3.1	ND
Methyl butanoate	1.29	ND
Butyl butanoate	1.26	ND
Ethyl hexanoate	0.85	ND
3-methyl-, butyl butanoate	0.28	ND
1-Hexyl acetate	0.47	ND
Octyl butyrate	0.8	ND
Octyl acetate	0.82	ND
Butanoic acid, octyl ester	2.52	ND
n-Octyl 2-methyl butanoate	0.67	0.26
Octyl isovalerate	0.98	ND
1-Methyl butyl butanoate	0.31	ND
Benzyl acetate	0.2	ND
Ethylene glycol di-n-butanoate	0.99	ND
2-methyl-,1,2-dimethyl propyl butanoate	0.27	ND
Octyl hexanoate	0.96	ND
Benzyl butanoate	0.18	ND
2,2,4-Trimethyl-1,3-pentanediol di isobutyrate	0.32	2.5
Ethyl 3-hydroxy hexanoate	0.17	ND
Isopropyl myristate	0.24	1.4
Isopropyl palmitate	ND	0.37
Methyl palmitate	ND	0.24
n-Decyl acetate	ND	0.16
**Aldehydes**		
Octanal	0.45	ND
Nonanal	0.94	ND
Decanal	0.26	0.28
Benzaldehyde	0.15	ND
2-Nonenal	0.16	ND
**Alchocol**	ND	ND
2-ethylHexanol,	0.16	ND
1-Octanol	0.72	ND
Benzyl alcohol	0.32	ND
1-Dodecanol	0.24	0.07
1-Decanol	ND	0.13
Epiglobulol	ND	0.77
Farnesyl alcohol	ND	1.75
n-Pentadecanol	ND	0.29
**Acids**		
Butanoic acid	0.27	ND
Hexanoic acid	1.48	ND
Decanoic acid	0.4	ND
**Ketones**		
Trans-beta-Damascenone	ND	0.18
Alpha Isomethyl ionone	ND	0.38
1,3-dimetylcyclohex-3-yl-ethyl-ketone	ND	4.8
Geranylacetone	ND	0.23
**Lactones**		
Gamma-Decanolactone	30.3	1.23
Gamma-Dodecalactone	8.16	5.23
**Terpenoids**		
Cis-Linalool oxide	0.15	0.52
Trans-Linalool oxide	0.7	0.34
Linalool	7.23	3.77
(E)-.beta.-Famesene	1.43	0.54
L-.alpha.-Terpineol	0.97	1.22
Alpha/beta.-Bisabolene	0.55	1.85
Trans-.alpha.-Bergamotene	0.18	0.14
Geraniol	0.48	0.4
Nerolidol	22.2	26
.Alpha.-Bisabolol	0.31	3.68
Nerol	ND	0.14
Alpha.-Bisabolol oxide	ND	7.18
Cis Bisabol-12-ol	ND	1.95
**Furans**		
Mesifuran; 4-methoxy-2,5-dimethyl-3(2H)-Furanone	0.67	ND
5-(1,5-dimethyl-1,4-hexadienyl)-2-ethenyltetrahydro-2-methyl -furan	0.81	12.2
5-(1,5-dimethyl-1,4-hexadienyl)-2-ethenyltetrahydro-2-methyl furan	0.79	16.5
Furaneol; 2,5-dimethyl-4-hydroxy-3[2H]-furanone	0.49	ND

* not detected.

**Table 2:** Effect of SPME method parameters on the peak area of compounds in strawberry samples.

		1	2	3	4	5	6
		60/15/15 **	60/15/15	80/15/15	60/15/15	60/30/15	60/30/30
Chemical Class	Compound	1 g Sample—No NaCl Added	5 g—No NaCl Added	5 g—No NaCl Added	5 g—NaCl Added	5 g—NaCl Added	5 g—NaCl Added
ester	Methyl butanoate	ND *	ND	738,282	1,315,683	707,398	830,957
ester	Ethyl butanoate	ND	ND	206,725	1,060,169	406,838	450,179
ester	Isopropyl butanoate	ND	ND	ND	267,049	ND	ND
ester	Methyl hexanoate	ND	68,855	ND	224,954	181,137	145,866
ester	Butyl butanoate	ND	256,712	85,848	561,824	95,841	59,241
ester	Ethyl hexanoate	ND	698,129	57,261	826,499	164,429	113,573
ester	Hexyl acetate	ND	156,936	46,806	202,327	99,420	63,675
ester	1-methylbutyl butanoate	ND	32,885	ND	25,673	31,605	ND
ester	Isopropyl hexanoate	ND	27,487	ND	48,863	ND	ND
ester	Butyl 2-methylbutanoate	ND	ND	ND	32,895	ND	ND
ester	Butyl isovalerate	ND	56,652	ND	66,437	ND	ND
furane	2,5-Dimethyl-4-methoxy-3(2H)-furanone (mesifuran)	ND	116,677	ND	225,405	86,645	72,332
alcohol	1-Octanol	71,907	111,901	ND	103,199	35,391	ND
monoterpene	Linalool oxide	25,514	16,361	44,240	29,820	34,715	ND
monoterpene	Linalool	423,746	476,308	692,883	763,605	687,195	501,058
ester	Butyl hexanoate	46,282	167,048	ND	ND	ND	ND
ester	Methyl octanoate	ND	ND	ND	18,472	17,033	ND
ester	Ethyl octanoate	ND	26,820	ND	ND	ND	ND
ester	Hexyl hexanoate	14,518	ND	44,537	225,887	ND	30,143
monoterpene	L-.alpha.-Terpineol	ND	ND	117,009	98,177	71,803	45,080
ester	Octyl acetate	86,302	529,189	82,090	315,916	124,460	63,368
ester	Methylbutyl isobutyrate	ND	ND	ND	38,437	ND	28,389
monoterpene	Nerol	ND	ND	ND	ND	ND	ND
monoterpene	Geraniol	ND	ND	47,063	26,609	70,671	16,849
alcohol	1-Decanol	46,194	23,315	25,954	37,982	ND	ND
ester	Nonanyl acetate	ND	27,434	ND	19,472	15,105	22,499
ester	Octyl isobutanoate	ND	26,276	177,820	10,348	ND	ND
ester	Hexyl hexanoate	ND	23,183	ND	ND	ND	ND
ester	Octyl butanoate	403,518	893,533	ND	678,059	167,528	79,103
ester	Decyl acetate	23,603	68,323	21,553	39,048	ND	12,517
ester	Linalyl butanoate	14,001	74,437	177,243	37,273	20,719	11,959
ester	Octyl 2-methylbutanoate	167,334	318,109	101,486	190,279	132,282	63,319
ester	Octyl 3-methylbutanoate	494,073	547,450	ND	228,347	192,827	87,736
ketone	trans-Geranylacetone	6009	14,190	39,572	14,751	26,223	17,848
sesquiterpene	beta.-Famesene	132,821	150,650	105,368	145,111	133,660	124,198
lactone	gamma.-Decalactone	2,961,011	3,159,451	2,378,656	3,665,747	3,788,141	2,401,025
sesquiterpene	alpha.-Bergamotene	ND *	39,914	26,378	ND	23,288	22,563
furane	5-(1,5-dimethyl-1,4-hexadienyl)-2-ethenyltetrahydro-2-methyl-furan	143,723	105,763	792,449	153,029	ND	195,733
sesquiterpene	alpha.-Farnesene	69,286	57,115	62,259	60,480	ND	59,104
furane	5-(1,5-dimethyl-1,4-hexadienyl)-2-ethenyltetrahydro-2-methyl-furan	211,162	135,746	101,2693	227,222	ND	289,369
sesquiterpene	beta.-Bisabolene	ND	ND	ND	25,982	ND	33,752
sesquiterpene	Nerolidol	4,129,991	4,293,677	511,0598	3,446,089	5,449,318	3,745,010
ester	Octyl hexanoate	220,594	332,034	48,661	169,774	48,289	19,301
ester	Decyl butyrate	ND	ND	ND	47,917	ND	ND
hydrocarbon	Hexadecane	91,956	22,169	35,650	23,703	ND	47,748
ester	Decyl-2-methylbutanoate	12,298	17,486	ND	10,367	ND	ND
ester	Decyl isovalerate	27,487	38,586	ND	16,267	ND	ND
sesquiterpene	cis-Bisabol-12-ol	ND	ND	222,004	ND	95,699	ND
furane	2-Furanmethanol	ND	ND	525,319	38,681	40,385	26,206
lactone	gamma.-Dodecalactone	932,616	1,222,723	2,661,323	1,586,906	2,395,865	1,247,452
sesquiterpene	alpha.-Bisabolol	ND	ND	705,355	199,679	260,118	108,620
ester	Nerolidyl acetate	53,232	149,778	197,391	199,895	216,020	70,630
sesquiterpene	Farnesol	ND	ND	256,784	ND	ND	ND
**total esters**		**1,549,795**	**4,443,636**	**1,788,312**	**6,704,870**	**2,404,911**	**2,081,825**
**total monoterpenes**		**535,562**	**1,021,858**	**866,276**	**1,135,950**	**917,041**	**581,275**
**total sesquiterpenes**		**4,332,098**	**4,541,356**	**6,231,962**	**3,877,341**	**5,962,083**	**4,093,247**
**total furans**		**354,885**	**358,186**	**2,330,461**	**644,337**	**127,030**	**583,640**

* not detected ** incubation temperature/incubation time/extraction time.

**Table 3:** GC-MS/MS parameters.

GC Parameters		
Start temperature	40 °C	
Temperature program		40 °C 5 min
	10 °C/min	230 °C 5 min
	10 °C/min	250 °C 5 min
Gas	Helium	
Flow rate	1 mL/min	
**MS parameters**		
Ion source	230 °C	
Interface	260 °C	
Detector voltage	0.8 kV	

**Table 4:** SPME experiment parameters.

PDMS Fiber	
Conditioning temperature	260 °C
Pre-condition time	5 min
Incubation temperature	40 °C/60 °C/80 °C
Incubation time	15 min/30 min
Sample vial depth	22 mm
Sample extract. time	15 min/30 min
Sample desorp. time	10 min
Post-condition. time	5 min
**DVB/CAR/PDMS Fiber**	
Conditioning temperature	260 °C
Pre-condition time	5 min
Incubation temperature	40 °C/60 °C/80 °C
Incubation time	15 min/30 min
Sample vial depth	22 mm
Sample extract. time	15 min/30 min
Sample desorp. time	10 min
Post-condition Time	5 min

## Data Availability

The raw data supporting the conclusions of this article will be made available by the authors on request.

## References

[1] Kafkas E., Kafkas S., Koch-Dean M., Schwab W., Larkov O., Lavid N., Ravid U., Lewinsohn E. (2005). Comparison of Methodologies for the Identification of Aroma Compounds in Strawberry. Turk. J. Agric. For..

[2] Flavourings | EFSA. https://www.efsa.europa.eu/en/topics/topic/flavourings.

[3] (PDF) The Composition of Strawberry Aroma Is Influenced by Cultivar, Maturity, and Storage. https://www.researchgate.net/publication/266228165_The_Composition_of_Strawberry_Aroma_Is_Influenced_by_Cultivar_Maturity_and_Storage.

[4] Strawberry Flavour Excipient | Uses, Suppliers, and Specifications. PharmaCentral Mater. Knowl. Platf..

[5] Annex to the European Commission Guideline on “Excipients in the Labelling and Package Leaflet of Medicinal Products for Human Use” | European Medicines Agency. https://www.ema.europa.eu/en/annex-european-commission-guideline-excipients-labelling-package-leaflet-medicinal-products-human-use.

[6] Strawberry: What Is It and Where Is It Used?. https://www.drugs.com/inactive/strawberry-116.html.

[7] Battino M., Giampieri F., Cianciosi D., Ansary J., Chen X., Zhang D., Gil E., Forbes-Hernández T. (2021). The Roles of Strawberry and Honey Phytochemicals on Human Health: A Possible Clue on the Molecular Mechanisms Involved in the Prevention of Oxidative Stress and Inflammation. Phytomedicine Int. J. Phytother. Phytopharm..

[8] Giampieri F., Tulipani S., Alvarez-Suarez J.M., Quiles J.L., Mezzetti B., Battino M. (2012). The Strawberry: Composition, Nutritional Quality, and Impact on Human Health. Nutr. Burbank Los Angel. Cty. Calif.

[9] STRAWBERRY: Overview, Uses, Side Effects, Precautions, Interactions, Dosing and Reviews. https://www.webmd.com/vitamins/ai/ingredientmono-362/strawberry.

[10] Mcfadden W.H., Teranishi R., Corse J., Black D.R., Mon T.R. (1965). Volatiles from strawberries. II. combined mass spectrometry and gas chromatography on complex mixtures. J. Chromatogr..

[11] Schieberle P., Hofmann T. (1997). Evaluation of the Character Impact Odorants in Fresh Strawberry Juice by Quantitative Measurements and Sensory Studies on Model Mixtures. J. Agric. Food Chem..

[12] Jetti R.R., Yang E., Kurnianta A., Finn C., Qian M.C. (2007). Quantification of Selected Aroma-Active Compounds in Strawberries by Headspace Solid-Phase Microextraction Gas Chromatography and Correlation with Sensory Descriptive Analysis. J. Food Sci..

[13] Abouelenein D., Mustafa A.M., Angeloni S., Borsetta G., Vittori S., Maggi F., Sagratini G., Caprioli G. (2021). Influence of Freezing and Different Drying Methods on Volatile Profiles of Strawberry and Analysis of Volatile Compounds of Strawberry Commercial Jams. Molecules.

[14] Ulrich D., Kecke S., Olbricht K. (2018). What Do We Know about the Chemistry of Strawberry Aroma?. J. Agric. Food Chem..

[15] Arthur C.L., Pawliszyn J. (1990). Solid Phase Microextraction with Thermal Desorption Using Fused Silica Optical Fibers. Anal. Chem..

[16] Parker J.K., Elmore J.S., Methven L., Woodhead Publishing (2015). Flavour Development, Analysis and Perception in Food and Beverages.

[17] Noguchi Y., Mochizuki T., Sone K. (2002). Breeding of a New Aromatic Strawberry by Interspecific Hybridization Fragaria x ananassa×F. Nilgerrensis. J. Jpn. Soc. Hortic. Sci..

[18] Bebek Markovinović A., Putnik P., Duralija B., Krivohlavek A., Ivešić M., Mandić Andačić I., Palac Bešlić I., Pavlić B., Lorenzo J.M., Bursać Kovačević D. (2022). Chemometric Valorization of Strawberry (Fragaria x Ananassa Duch.) Cv. ‘Albion’ for the Production of Functional Juice: The Impact of Physicochemical, Toxicological, Sensory, and Bioactive Value. Foods.

[19] Forney C.F., Kalt W., Jordan M.A. (2000). The Composition of Strawberry Aroma Is Influenced by Cultivar, Maturity, and Storage. HortScience.

[20] Morales-Quintana L., Ramos P. (2019). Chilean Strawberry (*Fragaria Chiloensis*): An Integrative and Comprehensive Review. Food Res. Int..

[21] Aharoni A., Giri A.P., Verstappen F.W.A., Bertea C.M., Sevenier R., Sun Z., Jongsma M.A., Schwab W., Bouwmeester H.J. (2004). Gain and Loss of Fruit Flavor Compounds Produced by Wild and Cultivated Strawberry Species. Plant Cell.

[22] Zellner B., Dugo P., Dugo G., Mondello L. (2008). Gas Chromatography-Olfactometry in Food Flavour Analysis. J. Chromatogr. A.

[23] Gomes Da Silva M.D., Chaves Das Neves H.J. (1999). Complementary Use of Hyphenated Purge-and-Trap Gas Chromatography Techniques and Sensory Analysis in the Aroma Profiling of Strawberries (Fragaria Ananassa). J. Agric. Food Chem..

[24] Lewers K.S., Newell M.J., Park E., Luo Y. (2020). Consumer Preference and Physiochemical Analyses of Fresh Strawberries from Ten Cultivars. Int. J. Fruit Sci..

[25] Zhang Z., Pawliszyn J. (1993). Headspace Solid-Phase Microextraction. Anal. Chem..

[26] Drakula S., Mustač N.Č., Novotni D., Voučko B., Krpan M., Hruškar M., Ćurić D. (2022). Optimization and Validation of a HS-SPME/GC–MS Method for the Analysis of Gluten-Free Bread Volatile Flavor Compounds. Food Anal. Methods.

[27] Sánchez-Palomo E., Díaz-Maroto M.C., Pérez-Coello M.S. (2005). Rapid Determination of Volatile Compounds in Grapes by HS-SPME Coupled with GC-MS. Talanta.

[28] Olbricht K., Grafe C., Weiss K., Ulrich D. (2008). Inheritance of Aroma Compounds in a Model Population of Fragaria × Ananassa Duch. Plant Breed..

[29] Ulrich D., Olbricht K. (2016). A Search for the Ideal Flavor of Strawberry—Comparison of Consumer Acceptance and Metabolite Patterns in Fragaria × Ananassa Duch. J. Appl. Bot. Food Qual..

[30] Vandendriessche T., Nicolai B.M., Hertog M.L.A.T.M. (2013). Optimization of HS SPME Fast GC-MS for High-Throughput Analysis of Strawberry Aroma. Food Anal. Methods.

[31] Abouelenein D., Acquaticci L., Alessandroni L., Borsetta G., Caprioli G., Mannozzi C., Marconi R., Piatti D., Santanatoglia A., Sagratini G. (2023). Volatile Profile of Strawberry Fruits and Influence of Different Drying Methods on Their Aroma and Flavor: A Review. Mol. Basel Switz..

[32] Padilla-Jiménez S.M., Angoa-Pérez M.V., Mena-Violante H.G., Oyoque-Salcedo G., Montañez-Soto J.L., Oregel-Zamudio E. (2021). Identification of Organic Volatile Markers Associated with Aroma during Maturation of Strawberry Fruits. Molecules.

[33] Cozzolino R., Amato G., Siano F., Picariello G., Stocchero M., Morra L., Mignoli E., Sicignano M., Petriccione M., Malorni L. (2022). New Biodegradable Mulching Films for Strawberry (Fragaria × Ananassa Duch.): Effects on the Volatile Profiles of the Fruit. Agronomy.

[34] Sheng L., Ni Y., Wang J., Chen Y., Gao H. (2021). Characteristic-Aroma-Component-Based Evaluation and Classification of Strawberry Varieties by Aroma Type. Mol. Basel Switz..

[35] González-Domínguez R., Sayago A., Akhatou I., Fernández-Recamales Á. (2020). Volatile Profiling of Strawberry Fruits Cultivated in a Soilless System to Investigate Cultivar-Dependent Chemical Descriptors. Foods.

[36] Urrutia M., Rambla J., Alexiou K., Granell A., Monfort A. (2017). Genetic Analysis of the Wild Strawberry (Fragaria Vesca) Volatile Composition. Plant Physiol. Biochem..

[37] Al-Taher F., Nemzer B. (2020). Identification of Aroma Compounds in Freeze-Dried Strawberries and Raspberries by HS-SPME-GC-MS. J. Food Res..

[38] Pyysalo T., Honkanen E., Hirvi T. (1979). Volatiles of Wild Strawberries, Fragaria Vesca L., Compared to Those of Cultivated Berries, Fragaria.Times. Ananassa Cv Senga Sengana. J. Agric. Food Chem..

[39] Molyneux R.J., Schieberle P. (2007). Compound Identification:  A Journal of Agricultural and Food Chemistry Perspective. J. Agric. Food Chem..

[40] Ozcan G., Barringer S. (2011). Effect of Enzymes on Strawberry Volatiles during Storage, at Different Ripeness Level, in Different Cultivars, and during Eating. J. Food Sci..

[41] Vandendriessche T., Vermeir S., Mayayo C., Hendrickx Y., Lammertyn J., Nicolaï B., Hertog M. (2013). Effect of Ripening and Inter-Cultivar Differences on Strawberry Quality. LWT—Food Sci. Technol..

[42] Suutarinen J., Honkapää K., Heiniö R.-L., Autio K., Mokkila M. (2000). The Effect of Different Prefreezing Treatments on the Structure of Strawberries Before and After Jam Making. LWT—Food Sci. Technol..

[43] Suutarinen J., Änäkäinen L., Autio K. (1998). Comparison of Light Microscopy and Spatially Resolved Fourier Transform Infrared (FT-IR) Microscopy in the Examination of Cell Wall Components of Strawberries. LWT—Food Sci. Technol..

[44] Bianchi G., Lucchi P., Maltoni M.L., Fagherazzi A., Baruzzi G. (2017). Analysis of Aroma Compounds in New Strawberry Advanced Genotypes. Acta Hortic..

[45] Flavor, Fragrance, and Odor Analysis. https://www.routledge.com/Flavor-Fragrance-and-Odor-Analysis/Marsili/p/book/9781138198579.

[46] Maarse H., van der Heij D.G. (1994). Trends in Flavour Research.

[47] Schulbach K.F., Rouseff R.L., Sims C.A. (2004). Relating Descriptive Sensory Analysis to Gas Chromatography/Olfactometry Ratings of Fresh Strawberries Using Partial Least Squares Regression. J. Food Sci..

[48] Williams A., Ryan D., Olarte Guasca A., Marriott P., Pang E. (2005). Analysis of Strawberry Volatiles Using Comprehensive Two-Dimensional Gas Chromatography with Headspace Solid-Phase Microextraction. J. Chromatogr. B Analyt. Technol. Biomed. Life Sci..

[49] Ruiz J., Quilez J., Mestres M., Guasch J. (2003). Solid-Phase Microextraction Method for Headspace Analysis of Volatile Compounds in Bread Crumb. Cereal Chem..

[50] Cosme F., Pinto T., Aires A., Morais M.C., Bacelar E., Anjos R., Ferreira-Cardoso J., Oliveira I., Vilela A., Gonçalves B. (2022). Red Fruits Composition and Their Health Benefits-A Review. Foods Basel Switz..

[51] Oz A.T., Baktemur G., Kargi S.P., Kafkas E. (2016). Volatile Compounds of Strawberry Varieties. Chem. Nat. Compd..

[52] King A., Readman J., Zhou J. (2003). The Application of Solid-Phase Micro-Extraction (SPME) to the Analysis of Polycyclic Aromatic Hydrocarbons (PAHs). Environ. Geochem. Health.

[53] Passa K., Simal C., Tsormpatsidis E., Papasotiropoulos V., Lamari F.N. (2023). Monitoring of Volatile Organic Compounds in Strawberry Genotypes over the Harvest Period. Plants.

[54] Furan, 5-(1,5-Dimethyl-1,4-Hexadienyl)-2-Ethenyltetrahydro-2-Methyl-, [2S-[2.Alpha.,5.Beta.(E)]]-SpectraBase. https://spectrabase.com/compound/6chDistPevs#stereoisomerCompounds.

[55] Castioni P., Kapetanidis I. (1996). Volatile Constituents from Brunfelsia Grandiflora Ssp. Grandiflora: Qualitative Analysis by Gc-Ms. Sci. Pharm..

[56] Martín-Del-Campo S.T., López-Ramírez J.E., Estarrón-Espinosa M. (2019). Dataset of Volatile Compounds Identified, Quantified and GDA Generated of the Maturation Process of Silver Tequila in New French Oak Barrels. Data Brief.

[57] Mahizan N.A., Yang S.-K., Moo C.-L., Song A.A.-L., Chong C.-M., Chong C.-W., Abushelaibi A., Lim S.-H.E., Lai K.-S. (2019). Terpene Derivatives as a Potential Agent against Antimicrobial Resistance (AMR) Pathogens. Molecules.

[58] PubChem Furaneol. https://pubchem.ncbi.nlm.nih.gov/compound/19309.

[59] Ducki S., Miralles-Garcia J., Zumbé A., Tornero A., Storey D.M. (2008). Evaluation of Solid-Phase Micro-Extraction Coupled to Gas Chromatography-Mass Spectrometry for the Headspace Analysis of Volatile Compounds in Cocoa Products. Talanta.

[60] Zhang R., Tang C., Jiang B., Mo X., Wang Z. (2021). Optimization of HS-SPME for GC-MS Analysis and Its Application in Characterization of Volatile Compounds in Sweet Potato. Mol. Basel Switz..

[61] Howard K.L., Mike J.H., Riesen R. (2005). Validation of a Solid-Phase Microextraction Method for Headspace Analysis of Wine Aroma Components. Am. J. Enol. Vitic..

[62] Ma Q.L., Hamid N., Bekhit A.E.D., Robertson J., Law T.F. (2013). Optimization of Headspace Solid Phase Microextraction (HS-SPME) for Gas Chromatography Mass Spectrometry (GC–MS) Analysis of Aroma Compounds in Cooked Beef Using Response Surface Methodology. Microchem. J..

[63] Ruiz-Hernández V., Roca M.J., Egea-Cortines M., Weiss J. (2018). A Comparison of Semi-Quantitative Methods Suitable for Establishing Volatile Profiles. Plant Methods.

[64] Noonan M.J., Tinnesand H.V., Buesching C.D. (2018). Normalizing Gas-Chromatography-Mass Spectrometry Data: Method Choice Can Alter Biological Inference. BioEssays News Rev. Mol. Cell. Dev. Biol..

[65] Schmarr H.-G., Bernhardt J. (2010). Profiling Analysis of Volatile Compounds from Fruits Using Comprehensive Two-Dimensional Gas Chromatography and Image Processing Techniques. J. Chromatogr. A.

[66] Ulrich D., Hoberg E., Rapp A., Kecke S. (1997). Analysis of Strawberry Flavour—Discrimination of Aroma Types by Quantification of Volatile Compounds. Z. Für Leb.-Forsch. A.

[67] Li X.-X., Fukuhara K., Hayata Y. (2009). Concentrations of Character Impact Odorants in ‘Toyonoka’ Strawberries Quantified by Standard Addition Method and PQ Column Extraction with GC-MS Analysis. J. Jpn. Soc. Hortic. Sci..

[68] Lin J., Dai Y., Guo Y., Xu H., Wang X. (2012). Volatile Profile Analysis and Quality Prediction of Longjing Tea (Camellia Sinensis) by HS-SPME/GC-MS. J. Zhejiang Univ. Sci. B.

[69] Fiehn O., Robertson D., Griffin J., van der Werf M., Nikolau B., Morrison N., Sumner L.W., Goodacre R., Hardy N.W., Taylor C. (2007). The Metabolomics Standards Initiative (MSI). Metabolomics.

[70] New in JMP 16 and JMP Pro 16 | JMP. https://www.jmp.com/en_is/events/mastering/topics/new-in-jmp16-and-jmp-pro16.html.

